# Once-nightly sodium oxybate (FT218) in the treatment of narcolepsy: a letter to the editor commenting on the recent publication by C. Kushida et al

**DOI:** 10.1093/sleep/zsab292

**Published:** 2022-06-13

**Authors:** Franck Skobieranda, Shawn Candler, Wayne Macfadden

**Affiliations:** Clinical Development, Neuroscience, Jazz Pharmaceuticals, Philadelphia, PA, USA; US Medical Affairs, Neuroscience, Jazz Pharmaceuticals, Philadelphia, PA, USA; Global Medical Affairs, Neuroscience, Jazz Pharmaceuticals, Philadelphia, PA, USA

Dear Editor,

We read with interest the publication titled “Once-Nightly Sodium Oxybate (FT218) Demonstrated Improvement of Symptoms in a Phase 3 Randomized Clinical Trial in Patients With Narcolepsy,” by C. Kushida et al., which was published recently in *Sleep* [1]. The authors present the results of the REST-ON trial of the investigational compound FT218, a once-nightly formulation of sodium oxybate (SXB), for the treatment of narcolepsy. We commend the investigators and sponsors of this study for their efforts to improve the lives of patients with narcolepsy and recognize the sensibility of working with the transformative compound oxybate. SXB (Xyrem) and lower-sodium oxybate (LXB; calcium, magnesium, potassium, and sodium oxybates; Xywav) are approved in the United States (US) for the treatment of excessive daytime sleepiness or cataplexy in patients 7 years of age and older with narcolepsy; LXB is also approved for the treatment of idiopathic hypersomnia in adults [2, 3]. We wish to comment on the authors’ interpretation of the REST-ON trial data and conclusions regarding the comparative efficacy and safety of FT218 with other oxybate formulations, as clarity on these matters is of great importance for patients and healthcare professionals.

First, it is important to note that the REST-ON trial did not involve a true parallel design, in which participants are assigned to groups exposed to different interventions or different doses of the same intervention. Instead, participants were randomized to receive placebo or escalating doses of FT218 (4.5 g [week 1], 6 g [weeks 2–3], 7.5 g [weeks 4–8], and 9 g [weeks 9–13]). Because of this design, conclusions based on within-study comparisons between FT218 doses (e.g., that the efficacy of 7.5 g was “nearly comparable to the 9-g dose”) are not appropriate. Moreover, the design of REST-ON effectively enriches for efficacy and tolerability at each step in the dose escalation; this enrichment further reduces the tenability of comparisons between FT218 doses.

Second, although Kushida et al. caution against cross-trial comparisons—a sentiment with which we agree—they nevertheless compared the efficacy and safety findings for FT218 in the REST-ON trial with SXB and LXB, drawing from multiple sources including the product labels and previous clinical trials. For example, the authors assert that lower doses of FT218 (6 g and 7.5 g) were associated with a significant benefit on excessive daytime sleepiness measured by the Epworth Sleepiness Scale compared with SXB (9 g/night). This assertion is based on observations from a clinical study published in 2002 that involved a different design from the REST-ON trial (ie, a true parallel-group design) with a smaller sample size (*n* = 28 who received SXB 9 g/night and completed the study, compared with *n* = 97 and *n* = 88 who completed the 6-g and 7.5-g epochs, respectively, in REST-ON), differences in baseline characteristics (58.1% female, 91.2% White, and mean age of 43.1 years, compared with approximately 68% female, approximately 75% White, and mean age of approximately 31 years in REST-ON), and shorter treatment duration (4 weeks compared with 13 weeks in REST-ON) [4]. Beyond this comparison with the 2002 SXB study, the markedly different designs of the REST-ON trial and pivotal studies of SXB or LXB preclude any meaningful cross-study comparisons.

Third, similar concerns exist with the authors’ statements regarding the relative safety of once-nightly versus twice-nightly oxybate. Kushida et al. reported adverse drug reactions (ADRs) for FT218 from the REST-ON trial and compared them at times to treatment-emergent adverse events (TEAEs) reported in clinical trials of SXB and LXB. ADRs are adverse events assessed by the investigator to be related or possibly related to study drug, whereas TEAEs reflect a broader category of all events reported by participants (whether or not related to study drug). In short, direct comparison of rates of ADRs and TEAEs is not appropriate. Furthermore, the authors state that falls related to treatment may be less likely with once-nightly dosing of oxybate compared with twice-nightly dosing. Although falls while receiving oxybate treatment have been reported, including in the REST-ON study, no basis exists to attribute such falls to a second nightly dose. In fact, getting out of bed after any oxybate dosing is discouraged, as noted in the publication and in the United States Prescribing Information (USPI).

Last, regarding the authors’ discussion about the efficacy and safety of oxybate as a function of dose frequency, evaluation of the oxybate literature as a whole, including studies of gamma-hydroxybutyrate [5], SXB [4, 6, 7], LXB [8], and FT218 [1], indicates a clear correlation between total daily exposure and both the (desirable) therapeutic effects and the (undesirable) adverse effects of therapy. In the absence of adequate and well-controlled head-to-head studies, it is challenging to differentiate efficacy, safety, or tolerability based on dose frequency. The case of improved safety of LXB lies in clear contrast, in which the improved safety profile compared with SXB, based on substantially reduced sodium content, was recognized by the US Food and Drug Administration [9] and is grounded in the expansive literature on global sodium intake and potential health consequences of exceeding recommended sodium intake levels.

We hope that our comments are received in the spirit of advancing the practice of sleep medicine with therapeutic use of oxybate in the treatment of narcolepsy.

## Data Availability

All relevant data are provided within the manuscript.
